# 

**Published:** 1875-09

**Authors:** 


FOUR DOLLARS A YEAR, POSTAGE FREE.

VOL. XXXII. September, 1875. NUMBER 9.

### THE Chicago Medical Journal AND EXAMINER.

Official Organ of the Chicago Medical Press Association.

ISSUED TWELVE TIMES A YEAR.

S.G.O. INDEXED

FOLIA SUA AD SANITATEM GENTIUM

**EDITOR:**

WILLIAM H. BYFORD, A.M., M.D.

**ASSOCIATE EDITORS:**

JAS. H. ETHERIDGE, M. D.
NORMAN BRIDGE, M.D.
JAS. NEVINS HYDE, A.M., M.D.
FERD. C. HOTZ, M.D.

TERMS—FOUR DOLLARS PER ANNUM, IN ADVANCE.

CHICAGO:
W. B. KEEN, COOKE & CO., Publishers,
NOS. 113 AND 115 STATE STREET.

COPYRIGHT — W. B. KEEN, COOKE & CO., A.D. 1875.

